# Book Reviews

**Published:** 1826-12

**Authors:** 


### CRITICAL ANALYSES.

Quæ laudanda forent, et quæ culpanda, vicissim

Illa, prius, cretâ; mox hæc, carbone, *notamus*.—PERSIUS.

*Medical Essays.—Essay first, on the Effects of Intestinal Irritation. Essay second, on some Effects of Loss of Blood. Essay third, on Exhaustion and Sinking from various Causes.* By MARSHALL HALL, M.D. F.R.S.E.; Physician to the General Dispensary near Nottingham.—8vo. pp. 96. Longman and Co. London.

THE name of Dr. MARSHALL HALL, who has so considerably increased our stock of useful knowledge on various subjects, will ensure the attention of our readers to whatever proceeds from his pen. With the modesty which generally accompanies talent, the present interesting essays are presented to us as "mere sketches, requiring to be filled up;" and the author "hopes to be assisted by the observations and commentaries of others, which he earnestly solicits."

The first Essay is "on some of the Effects of Intestinal Irritation." In the opinion of Dr. Hall, there are some of these effects, of an acute and alarming character, which are not understood in practice, nor discriminated from other morbid affections of a totally different nature. In many instances, we are told that the case resembles acute phrenitis; and it is this form of the disorder to which the attention of the profession is particularly directed. "In other instances, the affection has assumed the character of inflammation of the intestines or peritoneum. Occasionally the seat and kind of pain have led to the suspicion of pleuritis, or attacks of palpitation have suggested the idea of disease of the heart." Sometimes two or more of these affections take their rise in succession; the first or second probably ceasing entirely before the subsequent attack is established. Thus an erroneous idea is frequently entertained of the *metastasis* of inflammation, or other morbid action, from one organ to another. To illustrate more clearly the precise nature and deceitful character of this wandering irritation, a case is detailed "from one of the most respectable journals of the day," which was regarded "by its intelligent author, and apparently by the editor, as affording an example of migratory inflammation, or metastasis." The writer of the paper referred to is unknown to us, but he deserves honourable mention for the liberal feelings which induced him to permit the republication of it by Dr. Hall, who was avowedly opposed to the views that had been taken of the nature of the disease. We are aware of the difficulty of forming correct notions of cases which have not fallen under our own observation, however accurately they may be described; but, from mature consideration of the case commented upon, we think that we should not ourselves have carried the depletory system to such an extent.

Dr. Hall proceeds to state in detail the principal circumstances relative to the causes, symptoms, diagnosis, history, and treatment of this morbid affection; interspersing a few cases in illustration, in such a manner as to convey an idea of the gradual formation of his opinions.

"*The Causes.*—The principal cause of this morbid affection is a state of intestinal irritation of some duration, arising from a loaded condition of the bowels, or from a scybalous or disordered condition of their contents. But, although the presence of this cause appears essential to the production of the complaint, it is important to remark that I do not remember to have observed any example of it arising quite spontaneously from this cause *alone*. In every case there has been some superadded cause,—some shock sustained, or some extraordinary effort made on the part of the constitution, to rouse the dormant irritation into effect. Unusual fatigue, exertion, loss of rest, anxiety, or alarm,—a fall, or similar accident,—exposure to wet or cold,—any cause of weakness, and especially of exhaustion,—and particularly the combination of some of these circumstances always attendant on parturition, are the principal exciting causes of this affection. The patient has, in many instances, been subject to indigestion; and he is particularly liable to experience returns of the affection, in the same or some other form, until the primary disorder, and the consequent debility, be finally removed."

"*The Symptoms.*—This affection generally begins in the manner of a sudden attack. This attack is usually ushered in by rigor,—indeed by a more distinct and decided rigor than is observed in many cases of inflammation: the rigor is usually soon followed by much heat of surface; with the heat, the patient experiences some affection of the head, chest, or abdomen, and indeed, more or less, of all. There are vertigo on raising the head, pain, and some morbid impression on the mind,—panting in the breathing, and fluttering about the heart,—with general hurry, irritability, and restlessness; the tongue is white and loaded; the alvine evacuations are morbid, dark-coloured, fetid, and scybalous, or yellow like the yolk of egg, or of the appearance of yeast; the urine is turbid, and frequently deposits a copious sediment." (P. 7.)

To afford a more precise sketch of the symptoms of this affection, six cases are detailed.

With regard to *diagnosis,* the cases given are considered sufficient to establish the fact that there are attacks which resemble inflammation of the head, chest, or abdomen, and yet which are totally different in their nature. The author first observes, that—

" The attack from intestinal irritation is, in general, more sudden than that of inflammation, which is generally formed somewhat more gradually. This circumstance must, therefore, be cautiously inquired into, and may assist the diagnosis.

" I believe, too, that the seizure in the former case is attended by more distinct rigor, and afterwards by greater heat, than in the latter.

" The case of intestinal irritation affects, in a marked degree, more organs at once than that of inflammation, which is usually confined, at first at least, to one.

" The state of the tongue, and the condition of the alvine evacuations, are far more marked by disorder, and the latter are far more offensive, in attacks from intestinal irritation, than in cases of inflammation.

" The affection of the *head* from intestinal irritation comes on suddenly, is formed all at once, and is attended by great restlessness, suffering, and distress. In phrenitis, the disease is usually formed somewhat more gradually: the patient has been subject to pain in the head, perhaps, for some days, or even longer; he complains less, or at least there is less urgent distress,—less distress of a general kind; the pain may be very severe, although it is more frequently rather obscure; the intolerance of light and sound is less urgent; the rigor and subsequent heat, and the attack in general, are less marked; the patient is not so soon relieved by remedies; and the tongue and alvine evacuations are less morbid. In the attack of affection of the head from intestinal irritation, the patient is relieved, perhaps completely, if the lancet be employed, but the attack soon recurs with equal or greater violence: in phrenitis, the relief is seldom so complete, the interval of ease so long, or the return so marked; the pain is diminished, perhaps, but gradually resumes its former violence, unless active measures be interposed.

" When the *chest* is affected from intestinal irritation, the pain is severe and acute, and increased by a full inspiration; if the inspiration be repeated, however, a second and a third time, the increase of the pain is less and less. The situation of the pain varies; there is no cough, and no crepitus on making a full expiration. In all these respects the case differs from inflammation. The remarks already made respecting the relief from remedies, the tendency to a sudden recurrence of the pain, &c. in cases of affection of the head, apply equally here.

" I had long remarked that there might be both acute pain and tenderness under pressure of the abdomen, without inflammation; this state of things is frequently the result of intestinal irritation. It is distinguished from inflammation by the general symptoms of this affection, the mode of attack, the effects of remedies. In inflammation, the surface is usually cool, the head unaffected, the patient remarkably quiet: in the case of intestinal irritation, on the contrary, there is generally much heat after rigor, the head is much affected, and the patient is restless and generally distressed; the tongue is loaded, and perhaps swollen ; the alvine evacuations are extremely morbid, and great relief is obtained by the free operation of medicine." (P. 24.)

The mode of *treatment* comprises the full evacuation of the bowels, soothing by anodynes, light nourishment, and certain local remedies. "If (says the author) our diagnosis was early and certain, *perhaps* the lancet would never be required." There are two reasons which induce him to think this remedy ought not to be discarded entirely, even in cases of intestinal irritation. First, that which was originally irritation merely, may doubtless lead to a state of inflammation; the presence of much morbid fæces in the bowels may not only irritate and induce pain of that and of some remote part, but, if long continued, may eventually give rise to inflammation, and the lancet may be requisite as a *preventive,* if not as a cure. This observation applies especially to the attack of pain and tenderness in the abdomen; and much less so, our author thinks, to the affection of the head. Secondly, in the case of intestinal irritation, the diagnosis may not, until the symptoms of the affection be still further studied, be such as to remove all doubt as to the nature of the disease. It will then be prudent to bleed, for the sake of safety, whilst we enforce the other and more specific modes of treatment. After the bowels have been freely opened, the symptoms may still continue, partly from irritation produced by the purgatives, and partly from the lowness and exhaustion induced. To remove these effects, a draught of Træ. Opii and Spiritus Ammon. Arom., and light fluid nourishment, are recommended. "The local applications are, chiefly, a cold lotion applied to the head, a liniment to the chest, and a fomentation and liniment applied to the abdomen, when the pain occupies one or other of these parts."

Dr. Hall reserves it for future opportunities to pursue this interesting investigation. He candidly acknowledges that an accurate diagnosis is still required; and, indeed, every practitioner must be fully aware of the great difficulty which not unfrequently exists in distinguishing mere irritation, from whatever cause it may proceed, from actual inflammation. If we were called to a patient who, as in the sixth case given by Dr. Hall, "had lost nearly a gallon of blood," in whom the pain, having ceased in the part originally attacked, returned again with violence, "but moved to the right breast, and afterwards to the back," and whose motions were dark and fetid, we should certainly think the lancet had already had a fair trial, and adopt the method he judiciously advises,—viz. the combination of purgatives and sedatives. Where, however, there is a doubt as to the precise nature of the attack, (and doubts there will frequently be,) we would earnestly caution the practitioner against the dangerous error of endeavouring to relieve the local pain by the administration of opiates, to the exclusion of blood-letting; a method by which the disease is too apt to be masked, without being overcome.

The second Essay, "on some Effects of Loss of Blood," is reprinted from the Transactions of the Medical and Chirurgical Society, and has been noticed in a previous Number: it merits attentive consideration.

The third treats of "Sinking and Exhaustion from various causes." This subject, like those which precede it, has, in the opinion of our author, been too much neglected by medical writers. Some interesting observations on it have been made by JOHN HUNTER* and Sir H. HALFORD,† which are frequently referred to by Dr. Hall.

Our author considers sinking and exhaustion in relation, first, to early infancy; secondly, to old age; thirdly, to several diseases; and fourthly, to certain causes of exhaustion.

In early infancy, it is well known that exhaustion is very apt to be induced, and, "as the reaction is feeble at this period of life, the case soon assumes the character of sinking." Dr. Hall has "frequently been consulted when the original disease has been subdued, and the chief complaint of the little sufferer was a state of exhaustion, which a truce from remedies and medicine, and a proper supply of nourishment, and perhaps stimulants, have removed." This fact ought undoubtedly to be firmly impressed upon the minds of practitioners; and with this view we have adverted to it upon more than one occasion. Nothing, we are convinced, is more common, with inexperienced men, than to bleed and purge a child for the purpose of relieving a train of symptoms which is produced by the exhausting effects of these very remedies.

"When (says Dr. Hall) a child has been rather long ill, when active remedies have been employed, when the form of the disease has perhaps changed in some degree, and paleness of the cheeks

* Treatise on Inflammation, Part II. chap. ix. sect. 3.
† Transactions of the College of Physicians, vol. iv. p. 316, and vol. vi. p. 398.

is attended with irritability and restlessness, we should carefully consider whether the symptoms are not those of exhaustion. I am persuaded that, by relinquishing all lowering remedies, and adopting a cordial and soothing plan of treatment, I have seen some children recover, who would soon have sunk under the continuance of remedies calculated to subdue a supposed state of inflammation within the head, chest, or abdomen. In these cases, the idea that the original disease and the remedies had worn out the little patient, and led to a state of exhaustion, had apparently never occurred to the practitioner. It is impossible to do justice to this subject in a short section of a short essay; but I am persuaded that the hints here offered will, if carefully considered and cautiously acted upon, be of great assistance to the young physician in his treatment of some of the diseases of infants." (P. 75.)

In old age, as in infancy, the state of sinking may supervene, unaccompanied by symptoms of reaction: it is this state which has been described with so much accuracy by Sir H. Halford. The state of sinking in certain diseases is thus described:

"Some diseases are apt to issue, even at a rather early period, in a state of sinking; in other cases, sinking supervenes in the later stages of these diseases. This state seems sometimes to be the result of a direct influence of the disease in lowering the vital powers; sometimes the disease has subsided, but the state of sinking has continued and destroyed the patient; and sometimes the sinking has appeared to annihilate the morbid actions which constituted the disease, and thus to prove a *cure,* though a fatal one. In the latter cases, the physician, whose eye is fixed on the disease alone, and the friends of the dying patient, are apt, from the apparent truce in the actions or pains of the disease, to be led into a sanguine, though delusive, hope that the patient is better: there is, perhaps, a degree of dozing, mistaken for a long wished-for sleep; or some painful sensation has subsided, and the patient expresses himself as easier." (P. 82.)

The diseases in which the state of sinking becomes most marked, according to Dr. Hall, are typhus fever, enteritis, dysentery, and cholera. We have seen several cases of enteritis, in which, all the symptoms of the disease having subsided, and the patients have died suddenly, without mortification of any part of the bowels, or any other appearance explanatory of the phenomenon being detected on examination. It is invariably the rule of Sir H. Halford "still to consider the patient's life as in jeopardy until the intestines shall have performed their functions again, all irritation having left the stomach, and the skin remaining universally and equally warm."—Dr. Hall makes the following important observation connected with this subject:

"The state of sinking has not, I am persuaded, been distinguished from those forms of disease which have lately been more particularly attended to, and denominated *congestive;* yet the diagnosis is of the utmost moment, for, under the idea of congestion, the lancet has sometimes been used when stimuli were required to obviate a state of exhaustion or sinking." (P. 90.)

LAENNEC frequently refers to this state of sinking in cases of pneumonia. He remarks, that death is often caused more from the sinking of the vital principle than from the intensity or extent of the local affection.

In two cases of puerperal affection of the abdomen, mentioned by Dr. Hall, the patients sank rapidly. On examination, no morbid appearances whatever were found in either.

Dr. Hall concludes the volume with a few lines upon the subject of exhaustion from protracted lactation and profuse leucorrhœa. He believes that the former frequently gives rise to the latter; and that protracted lactation induces cough, with expectoration and wasting, leading to a particular form of consumption, of which he purposes to publish an account hereafter. We have seen several well-marked cases of this nature; but we refrain from dwelling particularly on the subject until we have the advantage of Dr. Hall's opinions in a more detailed form.

---

*Répertoire général d'Anatomie et de Physiologie, Pathologiques, et de Clinique Chirurgicale; ou, Recueil de Mémoires et d'Observations sur la Chirurgie, et sur l'Anatomie et la Physiologie, considerées dans les tissus sains et les tissus malades.* No. II. —Paris, 1826.

IN our July Number, we gave an abstract of the most interesting communications contained in the first part of this new French periodical, and we promised at the same time to bestow as much attention upon each succeeding part as the importance of its contents, and the known ability of the contributors, might appear to demand.

The first article of the Number before us is by M. A. RAIKEM, M.D. &c. The subject of it is one to which the French pathologists have lately paid very considerable attention—viz. *Inflammation and Softening of the Brain.* The memoir consists of a series of twenty-six cases; of a comparison between them, and of many analogous facts with which experience has furnished the author; of a brief sketch of the softening and hardening of the brain; and, lastly, a *coup d'œil* upon the connexions which exist between injuries of certain parts of the encephalon and the concomitant symptoms. The second case is short, yet interesting.

"A woman, sixty years of age, thin and pale, had been hemiplegic for six months, when she was sent to the Hôpital St. Antoine at Paris. The paralysis was attended by stiffness, and affected the right side. At the end of five months the disabled limbs gradually recovered nearly all their mobility and sensibility. The head was clear, and the intellectual faculties had suffered no remarkable alteration. The patient, however, was not entirely cured: she could not move either with perfect freedom or facility; and in this state she was attacked by a severe continued fever, which was *unaccompanied by any comatose affection or by headache*, but which shortly destroyed her.

"Upon examination, the head presented the following appearances:—The pia mater was infiltrated with serum; the right lateral ventricle contained four or five ounces of limpid fluid. The internal surface of the hemisphere of the brain, at the posterior part, above the cul de sac of the ventricle, was of a yellow colour; and a kind of cavernous ulcer was found in the cortical substance, the bottom of which was formed by a yellow pultaceous matter, similar to thickened pus."

This is one of the many instances upon record in which the appearances detected upon dissection could not have been anticipated by the most watchful attention to the symptoms exhibited during the life of the patient. If the subject of this case had, at the time of her decease, suffered from severe head affection, or if she had been comatose, the above-mentioned morbid appearances would have been regarded, even by the most skilful pathologists, as the cause of those symptoms. The progressive decline of the paralytic symptoms must have led to the belief that no serious mischief was going on in the head.

The third, fourth, fifth, and sixth cases, are instances of paralysis occurring suddenly, and terminating fatally. In each of these cases, fluid was found in the ventricles of the brain. In the third, the substance of the brain was preternaturally hardened. In the other cases, the cerebral mass was much softened in different parts, and a portion of its substance lost by ulceration.

Case VII.—A countryman, seventy years of age, above the middle stature, with an ample chest, well made and stout, was admitted in the summer of 1809. He had been affected for several months by symptoms which appeared to denote the existence of a passive dilatation of the right cavities of the heart. The face was swoln; the lips of a bluish cast; turgescent state of the external jugular veins; long-continued palpitations of the heart, which were manifest at the inferior part of the sternum. The slightest effort caused dyspnœa; the inspirations were very short. The inferior extremities were œdematous. A muscular weakness of the limbs of the left side gradually took place, in addition to the above symptoms. The head was unaffected; the intellectual faculties undisturbed, and the sensorial functions naturally performed.

At the end of a few days, the patient quitted the hospital at his own desire, but was again admitted during the winter of the same year. At this period the limbs of the left side were soft, flaccid, and motionless, but they still retained their sensibility. The symptoms of organic affection of the heart were now much aggravated. He was also affected with "*une fievre aiguë, muqueuse, ou gastro-enterique.*" During the last week of his life, he sank into so profound a state of lethargic stupor, that the animal functions appeared to be annihilated.

*Sectio cadaveris.*—The lower part of the inferior lobe of the right hemisphere of the brain contained several ounces of a white puriform liquid. At this part the substance of the brain, to a considerable extent, was hollowed into a cavity, the parietes of which, coated by a condensed purulent matter, were formed by the cerebral tissue, which was softened and pultaceous, very red at some points, and totally disorganised. The other parts of the brain were healthy. All the cavities of the heart, but especially those of the right side, were dilated. The ventricle of that side was large, and the ventriculo-auricular orifice also much enlarged. Several points of ossification on the circumference of the mitral valve, and upon the sigmoid valves of the aorta, were also observed. The small intestines were softer than natural.

The remaining cases principally consist of paralytic affections, accompanied by various trains of symptoms indicative of cerebral disturbance. Upon dissection, in most of them a softened state of some part of the brain was discovered, with serous effusion, or even small collections of matter.

In the twenty-first case, we have a curious instance of folly and credulity in the attendant of a child, labouring under symptoms which clearly indicated the existence of serious affection of the head, but which he mistook for a case of essential fever. He relied with much confidence upon the application of a pigeon divided in two portions, and applied palpitating to the head of the patient. The true nature of the case, and the fatal termination of it, were prognosticated by M. Raikem.

The second part of this memoir, which we presume (from what is said) will contain the results of the author's experience upon the connexion of particular symptoms with affections of particular parts of the brain, will doubtless be interesting, and shall not escape our attention.

The second communication is from M. Louis, upon the subject of *Abscesses of the Liver.*

It is presumed that much uncertainty still exists upon the subject of hepatic diseases, and, even with respect to inflammation of that important organ, many questions still remain to be determined. Some physicians still doubt, says M. Louis, whether abscesses of the liver, which contain laudable pus, can exist in the substance of the viscus, or whether they are situated only upon the surface, between it and the covering membrane. The doubt would long ago have been resolved, in the opinion of the author of the memoir, if sufficient attention had been bestowed upon the examination of all the viscera. Judging from his own experience, abscesses situated in the interior of the liver, and containing laudable pus, are not the least frequent occurrence. Five cases of this description have been detected out of 430 bodies which M. Louis has examined, and these are related in the present paper.

Whatever proceeds from the pen of M. Louis is entitled to our attention, but we are of opinion that, in the present day, no additional proofs are required to demonstrate the fact of the frequent occurrence of abscesses, containing laudable pus, in the internal parts of the liver. The voluminous work of Bianchi (Historia Hepatici), the size of which is in an inverse proportion to the quantity of useful matter it contains, maintains, it is true, a different doctrine. The united experience of the profession, however, has long ago set the subject at rest; and few practitioners of the present day are, we apprehend, even aware that it was ever contended that, if an abscess of the liver terminated favourably, it must necessarily have been confined either to the external surface of the liver, or to the peritoneal covering. It must be evident, however, that an abscess which occurs near or upon the surface, must be less dangerous than one which occupies a deeper seat.

We must pass over the detailed accounts of the cases, which appear to be faithfully described.

The general observations with which M. Louis concludes his paper are highly interesting. None of the patients suffered any pain in the right shoulder, and a doubt is expressed by the author whether this symptom, which is so strongly insisted upon by most writers upon the subject, really belongs to inflammation of the liver. It is suggested that, when it has occurred, there has been some concomitant affection of the lung or of the pleura of the right side, to which it ought to have been referred. Slight derangement of the liver, which is remediable by a purgative and abstinence for a day or two, is frequently the cause of a very painful sensation under the right shoulder. Of this fact we are convinced from the most certain, if not the most satisfactory, proof—*personal* experience. 1

It is a common opinion that a residence in hot countries favours the occurrence of hepatic disease: by many respectable authorities, however, this doctrine is disputed. It has also been lately asserted that, if hepatitis was not produced by external violence, it was always caused by inflammation of the mucous membrane of the duodenum. M. Louis does not deny that these two affections may exist at the same time, but his cases prove that such is not always the fact. In four of the five post-mortem examinations which he has recorded, the mucous membrane of the duodenum was found perfectly healthy: in the second case, it was a little softened, but without redness. Perhaps, says M. Louis, it may be thought extraordinary that in several cases we should have found the mucous membrane of the stomach and of the small intestines more or less inflamed, while the interjacent part, the duodenum, was unaffected. When, however, the mucous membrane of the stomach is inflamed, the inflammation generally stops at a certain distance from the pylorus; and when the mucous membrane of the small intestine is the seat of inflammation, it ordinarily commences in the portion nearest the cœcum, and rarely extends to the duodenum. The facts, therefore, reported, far from being extraordinary, are merely the expression of a general law.

Wounds of the head have been considered by authors to produce abscesses of the liver. M. Louis participates in the doubts entertained by Morgagni upon this subject. The abscesses of the liver which have been examined by M. Louis have generally been encysted. If the membrane lining the cavity of the abscess presented a firm appearance, it was presumed that it had been gradually formed. When the disease had pursued a rapid course, it was soft.

A softened state of the whole viscus is not admitted as a proof of inflammation having existed during life, unless the patient had suffered from the ordinary symptoms of hepatitis, or pus was discovered upon dissection. A softened state of many other organs is not incompatible with health. It is worthy of observation, that cicatrices are never seen in the substance of the liver. This fact is a proof of the danger at all times attending abscesses of this organ.

In four of the five cases related by M. Louis, jaundice existed; but still the biliary ducts transmitted the bile to the small intestines, even in a case in which a calculus of considerable size, arrested in the cystic duct, to a certain degree compressed the hepatic duct. M. Louis, indeed, has in no case been able to detect mechanical obstruction to have been the cause of jaundice.

The next paper is contributed by M. Marx: it relates the successful operation of tying the subclavian artery for aneurism of the left axillary artery, which was performed by Le Baron Dupuytren. We gave this case in our Hospital Reports last month.

*Memoir of the Anatomical Characters of Chronic Gastritis.* By M. Andral, fils. Second Part.

In the first part of this communication, which we have noticed in a former Number, the author has particularly described the various alterations which the mucous membrane of the stomach may undergo when it is attacked by inflammation. In this part he treats of the lesions of the other tissues which form the parietes of that viscus.

*On Morbid Invaginations of the Intestines.* By M. Dance, m.d.

In the first case, there existed invagination of the termination of the small intestine, of the cœcum, of the ascending and transverse colon, into the descending colon, so that the cœcum, which terminated the invagination, was placed in the sigmoid flexure of the colon. Gangrenous perforations were observed upon the parts forming the invagination. Peritonitis took place, and was rapidly fatal. The symptoms under which the patient laboured did not lead to any suspicion of the real nature of the case.

Case II.—Invagination of the small intestine, of the cœcum, and of the ascending colon, into the transverse and descending colon; the cœcum situated in the sigmoid flexure of the colon; disorganisation of the invaginated parts, and secondary peritonitis, which destroyed the patient.

The observations which follow the detailed relation of these cases do not differ, in any essential points, from the statements of other writers upon the same subject. In general, the symptoms which are produced by intestinal invagination are common to many other diseases. M. Dance offers a few observations upon the diagnosis, but he has not succeeded in detecting any symptoms of intus-susception which can relieve us from the difficulty with which we have had previously to contend. We may frequently suspect the existence of the disease, but we fear that a positive opinion can rarely be formed.

*Clinique Chirurgicale of the Hôtel Dieu.* By M. H. Royer Collard.

During the last three months, the operation of lithotomy has been performed five times in this hospital. Four cases terminated successfully; in two of which the lateral operation was performed by Breschet, and in the others Dupuytren performed the transverse operation, which has been described in the first Number of the Repertoire. In the fifth and fatal case, the recto-vesical operation was done by M. Sanson, in compliance with an arrangement which (as we mentioned in our September Number) these celebrated surgeons had determined upon, to give a fair trial to each mode of operating. The patient was sixty-five years of age, and of a broken-down constitution. An unsuccessful effort was made to crush the stone in the bladder by "*la méthode lithontriptique.*" Considerable irritation was produced by the attempt, from which, however, the patient perfectly recovered before he underwent the final operation, which was performed on the 13th May. The man died on the 13th of June.

M. Royer Collard adds some interesting observations upon the respective advantages and disadvantages of the different operations, as they are now performed at the Hôtel Dieu. In his opinion, the transverse or bilateral operation,* as it is now performed by M. Dupuytren, is not exempt from the hazards which have been feared from the other modes of operating. It is apprehended, also, that the serious accidents to which it exposes the patient would be frequent, if the operation were performed by any other surgeon than the Baron. The recto-vesical operation, as it is now performed by M. Sanson,† is thought by M. Collard to be the safest, although the patients recover from it more slowly than when the other modes of operating are adopted.

A brief description follows of various instruments which have been proposed by different individuals for the purpose of breaking down the calculus in the bladder, and afterwards extracting it without using the knife. Much ingenuity has certainly been displayed, and many difficulties have been surmounted. Small calculi we know may be removed by gradual dilatation of the urethra; but we have yet to discover the means of extracting stones of considerable size from the bladder, with less hazard and less suffering to the patient than is incurred by the skilful use of the knife.

The last article contains *the description of an apparatus, proposed by* M. Robinet, *to dissolve Calculi in the Urinary Bladder.* The difficulties to be overcome are—

* Vide our Number for September, p. 233 et seq.

† Ibid. p. 237.

1st. To grasp the calculus, and to enclose it in a pouch which is perfectly closed.

2d. To prepare a pouch capable of resisting the action of the liquids which must be employed to dissolve the calculus.

3d. To discover the best solvents for the various species of calculi, the time occupied in the dissolution, and the circumstances most favourable for its occurrence.

Much ingenuity is shown by M. Robinet in the conception of the proposed plan, and in the contrivance of the instruments. The description of the latter would be scarcely intelligible without the plates which are given in the accompanying Atlas, and to which we must refer our surgical readers. We hope that, upon trial, the plan may be found successful, but we fear it will not. Presuming even that a solvent is known which *will* act upon the calculus, and which will ***not*** act upon the pouch which contains it, we do not clearly understand by what means the operator is to get the calculus into the pouch. The utility of this contrivance has not yet been submitted to the test of experiment, either upon the dead or living body.

---

*The Morbid Anatomy of the Human Brain: being Illustrations of the most frequent and important Organic Diseases to which that Viscus is subject.* By Robert Hooper, M.D. Bachelor of Physic in the University of Oxford; Member of the Royal College of Physicians of London; Fellow of the Linnæan Society; Physician to the St. Mary-le-bone Infirmary, &c. &c. With coloured Plates.—4to. Longman and Co. London, 1826.

This is the most beautiful specimen of morbid anatomical plates that we have ever seen, and reflects the highest credit on the professional zeal and liberality of the author: professional zeal, because no one, who is not deeply interested in the advancement of the science, would devote to these investigations the time requisite for their accomplishment; and liberal, because it is well known that works of this expensive nature never become sources of private emolument; so it is but just to pay the tribute of acknowledgment and approbation to those who are disinterested enough to undertake them.

We are happy to learn from the Preface, that it is Dr. Hooper's intention to publish a series of engravings on a similar plan, which will comprehend delineations of the most important morbid appearances which the viscera of the human body assume in different diseases. We sincerely congratulate the public on the prospect of having this, which has long been acknowledged as a desideratum, so ably supplied; and we are convinced that the author will receive the approbation, as he is entitled to the gratitude of his professional brethren.

To convey any adequate idea of engravings by words, is as impossible as to give a satisfactory representation of the original appearances by any verbal description. The plates, therefore, must be seen to be appreciated, and all we can do is to describe the method adopted by the author in arranging the subjects.

The diseased appearances presented by the brain and its membranes are—inflammation and its effects,—tumors,—diseased structures and unnatural appearances, without tumefaction,—morbid collections of fluids secreted between the membranes and in the cavities,—extravasated fluids. The tumors are thus described:

"They are either,

"1. Aneurisma. The basilary and internal carotid arteries, or their branches, have been found aneurismal.

"2. Cephaloma. An organised, fungous, and vascular substance, mostly circumscribed, growing from a viscus, muscle, membrane, or nerve, and resembling the brain in appearance and feel. A cream-like fluid is easily squeezed from the cut surface. It is much less vascular than hæmatoma, and differs from it in appearance. Sometimes the natural structure of the organ is wholly destroyed, and converted into this disease.

"3. Chondroma. An organised, often excrescential substance, of the structure and hardness of cartilage. Tumors of this kind in the substance of the brain, though mentioned by good authorities, have not yet occurred to me; but I have often seen the glands of Pacchioni of this structure; and cartilaginous tumors in the dura mater are not uncommon.

"4. Hæmatoma. An organised, fungous, vascular substance, growing from a viscus, muscle, membrane, or nerve; and resembling, when cut, coagulated blood, with portions of a firmer texture, like the albuminous part of blood when solid. Sometimes the natural structure of the organ is wholly destroyed, and then the viscus is enlarged, and converted into this disease. In some instances it expands from a small peduncle, but it mostly has a broad base.

"5. Hygroma. A tumor formed by a collection of fluid, either serous, albuminous, or puriform, in the cellular membrane, or in a cyst. It is not uncommon to have a portion of the brain converted into a mass of cells, filled with a serous fluid. The pineal gland and the tunica arachnoides, especially about the medulla oblongata, occasionally present a circumscribed tumor which comes under this head, and hygroma often occurs as an encysted tumor in the substance of the brain.

"6. Melanoma. A soft, organised, fungous substance, of a black colour, mostly circumscribed and tubercular. The cut surface is smooth, of the colour of Indian ink; and very moderate pressure separates a fluid like the pigmentum nigrum of the eye. No part of the human body, in a healthy state, bears any resemblance to this diseased structure, except the gland-like bodies about the bifurcation of the trachea, which are occasionally very black, but very different in texture: it is therefore named from its colour, by which it is immediately known.

7. Osteoma. An organised, bony, or ivory-like tumor, found in the viscera and soft parts, consisting principally of phosphate of lime and a little animal matter, mostly circumscribed and excrescential, but sometimes tubercular. This genus includes those morbid secretions, and collections of phosphate and carbonate of lime, lithate of soda, and similar hard depositions, usually called calcareous.

"There is a diseased appearance that also comes under this head, which consists of a gritty calcareous deposition. It occurs in the very substance of the brain, and in the pineal gland, and consists principally of phosphate of lime and animal matter. These depositions are not larger than small particles of saw-dust; are mostly of an irregular form and spicular, and, when minutely examined with a lens, each portion is seen imbedded in the medullary substance, and attached to a blood-vessel. I have seen a cerebellum full of these, and the whole external surface studded with small spicular and bony particles immediately under the pia matral covering.

"8. Scrofula. The structure of the part is converted into one which answers to the definition of this disease, and which is sometimes circumscribed or tubercular, but more frequently not so. Encysted scrofulous tumors are met with.

"The encysted tumors are often seen adhering to the choroid plexus, resembling animal hydatids. These are mostly very small. Some are found imbedded in the medullary substance of the brain, from which in some cases they extend into the ventricles. These sometimes acquire a great size, and are generally so closely connected with the surrounding medullary substance as to require much nicety to pare it away, in order to expose the cyst; and in doing this many vessels are torn through which nourish the cyst, and secrete its contents.

"The cyst is formed of one membrane, which cannot be separated into laminæ. It is often of the thickness and texture of the dura mater, but in some remarkably thin. Those which are thick are very firm and opake; the thinner are more delicate, beautifully transparent, and have very much the appearance of animal hydatids. The vascularity of the transparent cysts is seen by vessels carrying red blood ramifying on their surface; but the vascularity of the opake cysts is not discernible by the naked eye.

"The contents of these encysted tumors are either a serous fluid, like that of the serum of the blood, or an albuminous fluid, or pus." (P. 12.)

The changes of structure and appearance without tumefaction are—flaccidity, firmness, pulpiness, and discoloration. The collections of fluid are either serous or purulent. The author states that he has found two ounces of a fluid, having every appearance of pus, between the dura mater and arachnoid membrane of the left hemisphere, without any vestige of inflammation of these membranes. There are fifteen plates in illustration of these various changes of structure.

---

*An Essay on Headaches, and on their Cure.* By WALTER VAUGHAN, M.D. of the Royal College of Physicians in London. —8vo. pp. 252. Longman and Co. London.

Dr. VAUGHAN has selected a subject which has puzzled the greatest masters of our art. We find, in many of our most esteemed writers, incidental confessions of the perplexities with which the general discussion of the many varieties of headache is entangled. As we were aware, then, of the difficulties to be encountered, we were not unreasonable enough to expect complete success. We sat down to the perusal of Dr. Vaughan's book with the most charitable feelings, but yet with the hopes of gleaning something from his labours which might repay us for our trouble, and prove acceptable to our readers. We have been disappointed.

The Preface to the work prepared us to meet with much uninteresting matter for a professional reader, as the author apologises for the introduction of "an analysis of SAUVAGES' Account of Headaches," which he would "have omitted if some friends, not of the profession, who had neither heard before of Sauvages, nor were aware of the imperfect state of our knowledge of headaches, had not resisted the omission." For the sake also of those friends, and of others who have not studied medicine as a science, he "has entered occasionally into such digressions, and such verbal criticisms, as the medical reader must see could not have been intended for him." The work, in fact, is neither one thing nor other; neither professional nor popular. The medical reader will find much that he does not require, and the popular reader much that he cannot understand.

If we are to judge from the numerous quotations with which the volume abounds, and which are introduced in the most unconnected manner, our author must have been a determined student. He refers to a most extensive range of literature,—from the Bible to ACCUM's "Death in the Pot." Dr. Vaughan has "never found" a patient stupid when affected with a headache: on the contrary, he has "always thought that, if he had received a liberal education, he could perform a mental analysis well, although he could not do so without an aggravation of his symptoms." Whoever completes the task which we have achieved, of perusing, *ab initio ad finem,* Dr. Vaughan's book, will probably be troubled with headache, and yet he will not find himself well prepared for the performance of "a mental analysis."

The style of a medical writer is but of secondary importance, provided the matter with which he furnishes us is useful. The faults of Dr. Vaughan's style, however, are certainly not redeemed by the excellence of his matter: the former is remarkably diffused and rambling; the latter principally consists of facts and speculative opinions, collected from various writers, and thrown together in a very heterogeneous manner.

To present our readers with an abstract of the contents of this book, would be impossible. We might have passed it over altogether, did we not consider it incumbent upon us, as reviewers, to point out those works which do not demand attention, as well as those upon which our readers may expend a portion of their time with advantage. Dr. Vaughan has "often lamented that the second edition of a book was inferior to the first." If the present Essay should pass to a second edition, which we do not apprehend it will, it would be difficult for the author to fall into the error himself which he laments in others: we cannot conceive that a second edition of it could be more unsatisfactory than its predecessor. Dr. Vaughan is fond of quoting Horace: he cannot, however, have read "De Arte Poetica" with attention; or, at least, he has not with profit. The following wholesome advice must have escaped his eye:

> "Sumite materiam vestris, qui scribitis, æquam
> Viribus; et versate diu, quid ferre recusent,
> Quid valeant humeri."

---

*Manual of Pathology; containing the Symptoms, Diagnosis, and Morbid Characters of Diseases: together with an Exposition of the different Methods of Examination, applicable to Affections of the Head, Chest, and Abdomen.* By L. Martinet, d.m.p. Resident Physician of the Hôtel Dieu. Translated, with Notes and Additions, by Jones Quain, a.b. Demonstrator of Anatomy at the Medical School, Aldersgate-street.—18mo. pp. 310. Anderson, London, 1826.

This little volume will be of use to the student: its extreme minuteness in the details, is its greatest fault; its extreme minuteness in size, its greatest recommendation.

The first part relates to the general method of examination applicable to different diseases, in which there is much useful information, diluted in a profusion of verbiage.

The second part treats of particular diseases, giving a brief sketch of the symptoms, diagnosis, and pathology of each. The first disease thus described is Fungus of the Dura Mater, and a general idea of the plan may be gathered from the following extract:

"*Fungus of the Dura Mater.—Symptoms:* This disease is of rare occurrence, but is not confined to any particular period of life. It may sometimes exist without occasioning any derangement of function, or, if it manifests any symptoms, they are so obscure as scarcely to indicate its existence. But after some time, probably during the progress of an old syphilitic taint, or in consequence of a contusion of the head, violent headaches occur, which may be either dull or lancinating, continued or intermittent, and occasionally accompanied by epileptic, comatose, or paralytic symptoms: at length a tumor begins to appear, the seat of which may be either at the roof or base of the brain, or sometimes in the orbit. This production is more or less hard, indolent or very painful, increases rather slowly, and exhibits a sort of pulsatory motion. It may at times be reduced altogether, or in part, within the walls of the cranium, and then we can distinctly trace the margins of the aperture through which it had escaped, which we find to be rough and irregular. Pressure, directed from above downwards on the tumor, gives rise to paralytic or comatose symptoms, for by this means it is made to compress the brain; but, if we press it from side to side between the fingers, no particular effect is produced, or at most only a slight degree of pain, for then no impression is made on the substance of the brain. Sometimes the cerebral symptoms ceases (cease) altogether after the tumor has escaped beyond the cranium.

"*The diseases with which it may be confounded.*—This affection may, in its first stage, be confounded with any of the derangements of the brain or its investments; in the second, with encephalocele, with vascular tumors of the dura mater following wounds, with abscess, with certain wens, or with aneurism of the occipital or temporal arteries.

"*Anatomical characters.*—These tumors are fibrous in their texture, sometimes crossed by enlarged blood-vessels; in some points they become softened and broken down, and contain blood effused into their substance. In some instances we find only one of them, in others several, which may be encysted, circumscribed, and more or less irregular. At first they are flattened before they escape beyond the skull, afterwards assume the form of a mushroom, the pedicle corresponding to the aperture in the cranium. The margins of the opening are eroded, and in many cases present asperities which, by pressing against the tumors, excite intense pain. (P. 120.)

The subdivisions of disease in this little volume are infinite, and such as never have been, and never can be, recognised during life. The pathology, as might be expected, is entirely French. The time will probably come when it will be thought quite as useful to distinguish diseases during life, and to prevent patients from dying, as to fill a museum with preparations made from them after death. We do not mean to undervalue morbid anatomy, but we are satisfied it is at present over-rated: we believe that a man may be intimately acquainted with all the changes of structure to be met with, and yet be ignorant of disease.

The translation seems to be well executed: the mistake in the preceding page, of course, is a typograghical error.

