# Errata

**Published:** 1827-01

**Authors:** 


					ERRATA.-
?In onrlast, paste 500, line 12 fiom the bottom, for " liberal'' read " liberality

				

